# Reconstruction of the Evolutionary Origin, Phylodynamics, and Phylogeography of the Porcine Circovirus Type 3

**DOI:** 10.3389/fmicb.2022.898212

**Published:** 2022-05-18

**Authors:** Yongqiu Cui, Lei Hou, Yang Pan, Xufei Feng, jianwei Zhou, Dedong Wang, Jinshuo Guo, Changzhe Liu, Yongyan Shi, Tong Sun, Xiaoyu Yang, Ning Zhu, Xinxin Tong, Yongxia Wang, Jue Liu

**Affiliations:** ^1^College of Veterinary Medicine, Yangzhou University, Yangzhou, China; ^2^Jiangsu Co-innovation Center for Prevention and Control of Important Animal Infectious Diseases and Zoonoses, Yangzhou University, Yangzhou, China; ^3^College of Animal Science and Technology, Anhui Agricultural University, Hefei, China

**Keywords:** porcine circovirus type 3, evolutionary origin, phylodynamics, phylogeography, reconstruction

## Abstract

Porcine circovirus type 3 (PCV3) is a newly identified virus associated with porcine dermatitis and nephropathy syndrome (PDNS) and multisystemic inflammatory responses in pigs. Recent studies suggests that PCV3 originated from bat circoviruses; however, the origin time, mode of spread, and geographic distribution of PCV3 remain unclear. In this study, the evolutionary origin, phylodynamics, and phylogeography of PCV3 were reconstructed based on the available complete genome sequences. PCV3 showed a closer relationship with bird circovirus than with bat circovirus, but their common ancestor was bat circovirus, indicating that birds may be intermediate hosts for the spread of circoviruses in pigs. Using the BEAST and phylogenetic analyses, three different clades of PCV3 (PCV3a, PCV3b, and PCV3c) were identified, with PCV3a being the most prevalent PCV3 clade. Further studies indicated that the earliest origin of PCV3 can be traced back to 1907.53–1923.44, with a substitution rate of 3.104 × 10^–4^ to 6.8524 × 10^–4^ substitution/site/year. A phylogeographic analysis highlighted Malaysia as the earliest location of the original PCV3, which migrated to Asia, America, and Europe. Overall, this study provides novel insights into the evolutionary origin, spread mode, and geographic distribution of PCV3, which will facilitate the prevention and control of PCV3 epidemics in the future.

## Introduction

Tracking the origin of emerging pathogens is crucial for controlling their spread, and research on the transmission of pathogens with different species helps prevent pandemics (Gao, 2018). The porcine circovirus (PCV) belongs to the genus *Circovirus* within the family *Circoviridae*. Before 2015, only two types of PCVs were known: porcine circovirus type 1 (PCV1) and porcine circovirus type 2 (PCV2) (Mankertz and Hillenbrand, 2002; Cheung, 2003). PCV1 was first detected in the porcine kidney cell line PK15 (ATCC-CCL31) in 1974, and is considered a non-pathogenic virus in pigs (Tischer et al., 1974; Allan et al., 1995). In 1991, PCV2 was first detected in Canada and was subsequently found in various countries around the world (Allan et al., 1995). PCV2 is linked to PCV-associated disease (PCVAD), including post-weaning multisystemic wasting syndrome, reproductive failure, respiratory disorders, and enteritis; PCV2 mainly damages the immune system of the host, thereby leading to immunosuppression in pigs (Firth et al., 2009; Denner and Mankertz, 2017; Jiang et al., 2019, 2020). PCVAD is now endemic in all pig-rearing countries and regions, causing huge economic losses in the pig industry worldwide. Using next-generation sequencing analysis, two novel porcine circoviruses were identified in 2015 and 2019: porcine circovirus type 3 (PCV3) and porcine circovirus type 4 (PCV4) (Palinski et al., 2016; Phan et al., 2016; Zhang H. H. et al., 2020).

In 2015, PCV3 was first identified in PCV2-negative sick pigs suffering from porcine dermatitis and nephropathy syndrome (PDNS) and reproductive disorders in the United States and was subsequently detected in many countries, including China, South Korea, Brazil, Poland, Thailand, Denmark, Spain, Germany, Japan, India, and Italy (Kwon et al., 2017; Stadejek et al., 2017; Fux et al., 2018; Hayashi et al., 2018; Kedkovid et al., 2018; Bera et al., 2021; Sun et al., 2021). PDNS-like clinical disease was successfully reproduced when administered experimentally to pigs with the PCV3 strain rescued from an infectious PCV3 DNA clone (Jiang et al., 2019). The PCV3-infected piglets showed clinical symptoms, including fever, diarrhea, coughing, sneezing, anorexia, lethargy, multifocal papules, and rubefaction on the skin and ears (Jiang et al., 2019, 2020). PCV3 infection can lead to severe disruption of the immune system, as evidenced by the presence of lymphocytic dysplasia and necrosis in many lymphatic tissues and organs in piglets. Currently, PCV3 infection has spread in most pig-rearing countries and regions worldwide, potentially causing great harm to the global pig industry.

The PCV3 genome consists of a circular single-standard DNA 2,000 bp in length that comprises two major open reading frames (ORFs): ORF1 encodes a replicase protein (Rep), which possesses 48% amino acid (aa) identity to the PCV2 Rep protein and is associated with viral replication; ORF2 encodes the capsid (Cap) protein, which is the major immunogen of circoviruses and shares only 37% amino acid identity with PCV2 Cap (Nawagitgul et al., 2000). The phylogenetic analysis is a powerful tool that has been applied in tracing the origin of viruses and analyzing their evolution, including those of PCV3 (Li et al., 2018). The earliest research, based on its limited sequence information, found that the time of the most recent common ancestor (tMRCA) was mainly concentrated in 2013 (Li et al., 2018). Afterward, with the increase in sequence information, other researchers re-analyzed the tMRCA time and found that the origin time may be concentrated around the 1950s (Chen et al., 2019). In a previous study, some researchers also conducted research on the geographical spread of PCV3 and found that China may be an important place for its spread (Franzo et al., 2019b).

The transmission of viruses within different species poses a potential threat to public safety. Some viral infections, including coronavirus and Zika virus infections have been demonstrated to adversely affect public health (Dye and Gay, 2003; Lazear et al., 2016; Zumla et al., 2016; Boni et al., 2020; Clausen et al., 2020). Circoviruses have been detected in humans, canines, birds, and cattle (Phenix et al., 2001; Hattermann et al., 2003; Li et al., 2010; Phan et al., 2016). In particular, PCV3 has been identified in mice, dogs, and other mammals (Phan et al., 2016; Zhang et al., 2018; Sun et al., 2019), indicating that PCV3 may exhibit a certain affinity for mammals. However, there is no evidence that PCV3 can directly infect humans.

In this study, we obtained PCV3 complete genome sequences from the NCBI database. Based on previous research, we increased the number of sequences to obtain more accurate information about the evolutionary origin, spread mode, and geographic distribution of PCV3. These findings are expected to aid the prevention and control of PCV3 epidemics in the future.

## Materials and Methods

### Sequence Datasets

All sequences used for this study were downloaded from the NCBI GenBank database^1^, and 616 PCV3 strains were obtained (accessed on August 31, 2021) (Supplementary Table 1). Furthermore, we downloaded 18 strains of bat circovirus, 9 strains of duck circovirus, 6 strains of human circovirus, 5 strains of bird circovirus, 7 strains of penguin circovirus, 6 strains of goose circovirus, 9 strains of canine circovirus, and 4 strains of fox circovirus.

The multiple alignments done via the fast Fourier transform (MAFFT) algorithm were used to compare and sort the complete genome and *Cap* gene used in this study (Zhang et al., 2018). SplitsTree and RDP4 software were used for recombination analysis (Martin et al., 2015), and recombination events were confirmed by at least four methods with a *p*-value cutoff of 0.05.

### Tracking the Origin of Porcine Circovirus Type 3

To investigate the tMRCA and evolutionary rates, we used Bayesian Markov chain Monte Carlo methods within Bayesian evolutionary analysis sampling trees (BEAST) (V1.10.4) (Drummond et al., 2012). The best substitution model, GTR + F + I + G4, assuming an uncorrected relaxed clock (lognormal), was selected using the ModelFinder software according to the Bayesian information criterion (BIC) score and according to the ps/ss (path sampling/stepping-stone sampling) values (Kalyaanamoorthy et al., 2017; Zhang D. et al., 2020). Three independent runs were performed with a chain length of 5 × 10^9^ generations, obtained every 10,000 generations, and combined using LogCombiner software. The results were estimated using the Tracer software (V1.7.1) after burn-in (10%). Parameters with an effective sampling size of more than 200 were accepted (Li et al., 2018). The final MCC tree was displayed using the FigTree software (V1.4.4).

To understand the relationship between different species of circoviruses, an ML tree was constructed with hosts of different species of the *Cap* gene using the RAxML software with the GTRGAMMAI model with 1,000 replicates. An MCC tree was reconstructed with complete genomic sequences to corroborate the ML tree.

Furthermore, discriminant analysis of principal components (DAPC) was used to analyze the genetic structure of populations from different countries. DAPC relied on the classical analysis of variance model, which was conducted as described by Lemey et al. (2019).

### The Bayesian Phylogeography of Porcine Circovirus Type 3

Bayesian stochastic variable selection (BSSVS) is typically used for spatial propagation (Lemey et al., 2019). According to the protocol, the BSSVS was allowed a Bayes factor (BF) and posterior probability (PP) test, which was used to identify the most reliable description of the spreading mode. A BF > 3 and PP > 0.3 can be considered as a significant migration route between country pairs. The BF of the migration rates and PP values were calculated using SpreaD3.

### Simulation of the Porcine Circovirus Type 3 Cap Protein Structure

The structures of the Cap protein were built by homology modeling, and suitable templates were selected for these protein sequences using the Swiss model^2^ for homology modeling (Waterhouse et al., 2018). PyMOL was used to generate model images.

### Selection Analysis of Porcine Circovirus Type 3 Cap Protein

Selection of the Cap protein of PCV3 was conducted using the DATAMONKEY program^3^. The methods utilized to estimate positive codon sites included fixed effects likelihood (FEL), fast unconstrained Bayesian approximation (FUBAR), single-likelihood ancestor counting (SLAC), and mixed-effects model of evolution (MEME). The positive selection sites were confirmed under positive selection by at least two methods with *p*-values for both FEL and MEME of less than 0.05, *p* < 0.1 by SLAC, and a posterior probability > 0.9 by FUBAR.

## Results

### Evolutionary Origin and Epidemiological Dynamics of Porcine Circovirus Type 3

**FIGURE 1:**
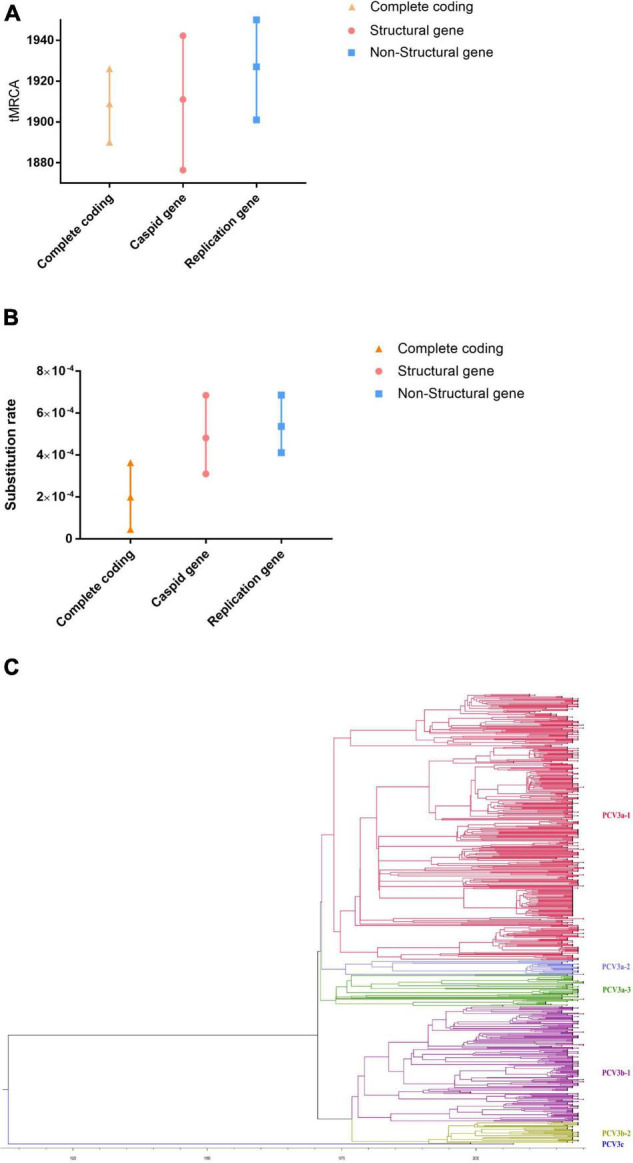
The tMRCAs and substitution rates were evaluated in BEAST (v1.10.4) for complete coding sequences and individual genes of PCV3. **(A)** The tMRCA of PCV3. **(B)** The substitution rate of PCV3. Yellow triangles represent the complete genome, red circles represent structural genes, and blue squares represent non-structural genes. **(C)** Phylogenetic analysis of the 616 *Cap* gene sequences. The MCC tree was reconstructed using BEAST (v1.10.4). The different clades are expressed by different RGB colors as indicated. The RGB color numbers were FF0033, 6666FF, 339900, 990099, 999900, and 170DF7, for PCV3a-1, PCV3a-2, PCV3a-3, PCV3b-1, PCV3b-2, and PCV3c, respectively.

RDP4 was used to detect recombination and there was no recombinational event existed among PCV3 strains. To investigate the time of the most recent ancestor (tMRCA) and the substitution rate, BEAST (V1.10.4) were used to estimate the *Rep* gene (ORF1), *Cap* gene (ORF2), and complete coding (ORF1 + ORF2) sequences. The tMRCA of PCV3 was 1911.97 with 95% highest probability density (HPD), ranging from 1876.43 to 1942.22 (Figure 1A). The tMRCA of the complete coding sequence, *Cap* gene, and *Rep* gene was 1907.53, 1911.97, and 1923.44, respectively (Figure 1A). Compared with previous studies, the earliest time of the PCV3 origin was traced to the beginning of the nineteenth century, which was earlier than what other researchers had speculated (Chen et al., 2019). The nucleotide substitution rate was 4.818 × 10^–4^ substitution/site/year with 95% HPD ranging from 3.104 × 10^–4^ to 6.8524 × 10^–4^ substitution/site/year (Figure 1B). The nucleotide substitution rates of the complete coding sequence, *Cap* gene, and *Rep* gene were 1.8989 × 10^–4^, 4.818 × 10^–4^, and 5.3707 × 10^–4^ substitution/site/year, respectively (Figure 1B). The substitution rate of PCV3 (Figure 1B) was slower than that predicted in a previous report (Li et al., 2018) or similarly to that predicted in other report (Chen et al., 2019).

As shown in Figure 1C, there are three different clades of PCV3 (PCV3a, PCV3b, and PCV3c). Moreover, PCV3a can be classified into three different subclades, PCV3a-1, −2, and −3, while PCV3b can be divided into two different subclades, PCV3b-1 and −2. To classify the PCV3 strains better, we classified the genetic distance. The major clade (PCV3a) contained most sequences and showed highly homogeneous sequences, with an average p-distance of 0.012, which ranged from 0.00 to 0.038. The average p-distance of clade 2 (PCV3b) was 0.013, which ranged from 0.00 to 0.024. PCV3c was the only strain that was detected in affected/dead pigs in an outbreak of highly pathogenic porcine reproductive and respiratory syndrome (PRRS) in Hunan province, China, in 2006, and the PCV3c genetic distance between PCV3a and PCV3b was high, both exceeding 0.114. PCV3a and PCV3b could be separated by a p-distance between 0.040 and 0.114, and if the p-distance was more than 0.114, it can be classified into the PCV3c clade. This result was also confirmed by DAPC analysis, which suggests that there are six different gene branches in the PCV3 viral population (Supplementary Figure 1).

Interestingly, we also found that the 289th nucleotide base of the *Cap* gene of PCV3 has a mutation; PCV3a and PCV3c contained G, while PCV3b contained A. However, this nucleotide change is a synonymous substitution and does not cause a change in the translated amino acid (proline), nor does it affect the virulence of the virus.

### Phylogeographic Analysis of Porcine Circovirus Type 3

**FIGURE 2:**
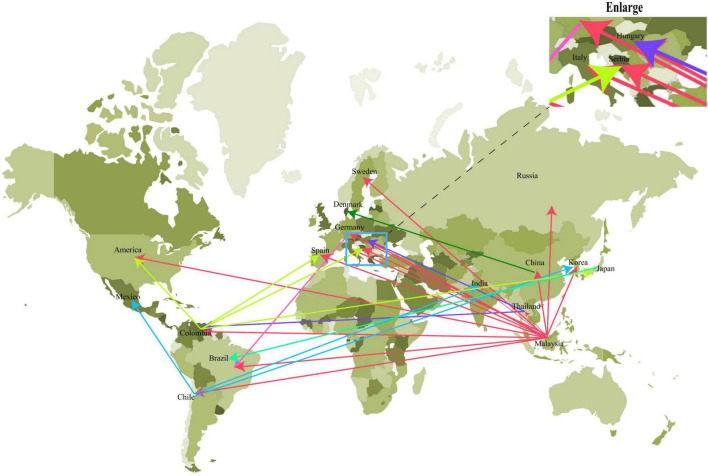
The Bayes factor test of the migration route of PCV3 based on BSSVS analysis. Only routes supported by a BF > 3 and PP > 0.3 were indicated. The migration status of different countries is indicated by arrows of different colors.

To eliminate the uncertainty in the estimation process, the relationships among the strains were estimated based on well-supported contacts (BF > 3 and PP > 0.3) among different countries. As shown in Figure 2, there are three main transmission areas: Asia, America, and Europe. Based on migration routes, we found that Malaysia was the most likely responsible for the spread of the virus to other countries, including Asia [China (BF = 91370.49), South Korea (BF = 29.65189), Thailand (BF = 91370.49), India (BF = 9.936503)], Europe [Russia (BF = 13037.49), Germany (BF = 11405.3), Spain (BF = 7.476306), Serbia (BF = 21.33482), Hungary (BF = 20.87616), Italy (BF = 14.09983)], America [United States (BF = 89.3468), Brazil (BF = 6.700879), Colombia (BF = 1432.32), and Chile (BF = 91370.49)] with high BF and transition rate (>0.9243) (Supplementary Table 2). Furthermore, there were other transmission routes in three continents, including countries such as Thailand, Colombia, and Chile (BF > 3 and transition rate > 0.9293) (details can be found in Supplementary Table 2).

### Relationship Between Multiple Species of Circoviruses

**FIGURE 3:**
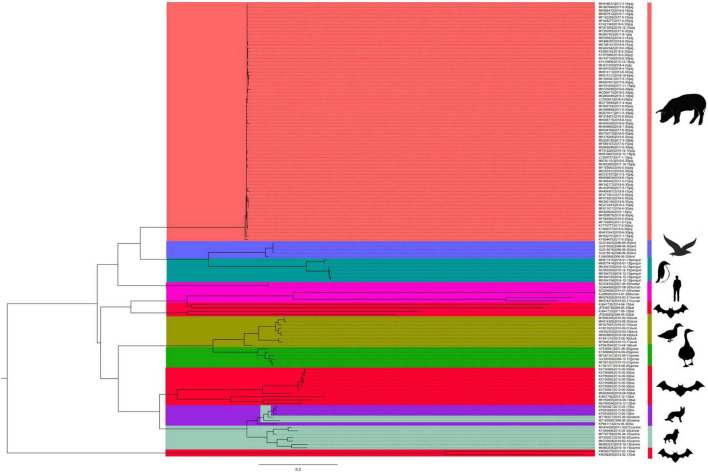
The origin of PCV3 was deduced using the complete genomes of the different circoviruses. MCC tree was reconstructed using BEAST (v1.10.4). The different circoviruses are expressed by different RGB colors as indicated. The RGB color numbers are FF6666, 6666FF, 009999, FF00CC, FF003C, 999900, 18A90A, 9926E0, and 99CBB8 for PCV3, bird circovirus, penguin circovirus, human circovirus, bat circovirus, duck circovirus, goose circovirus, fox circovirus, and canine circoviruses, respectively.

**FIGURE 4:**
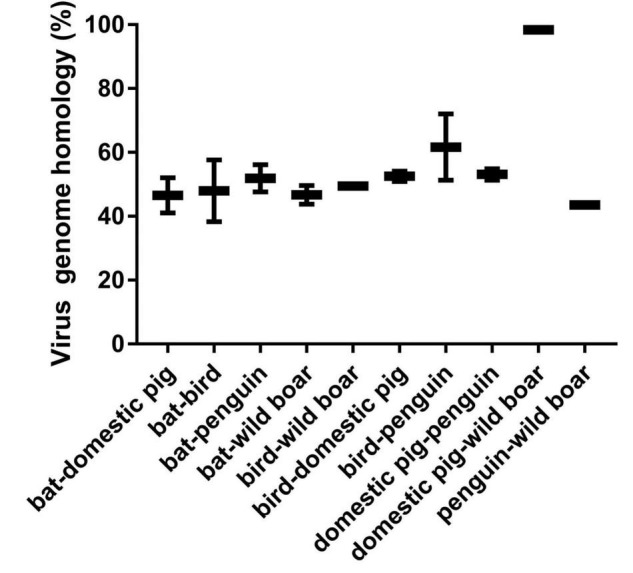
Comparison of homology of different species of circoviruses based on their complete genomes. Pig circovirus refers specifically to PCV3.

**FIGURE 5:**
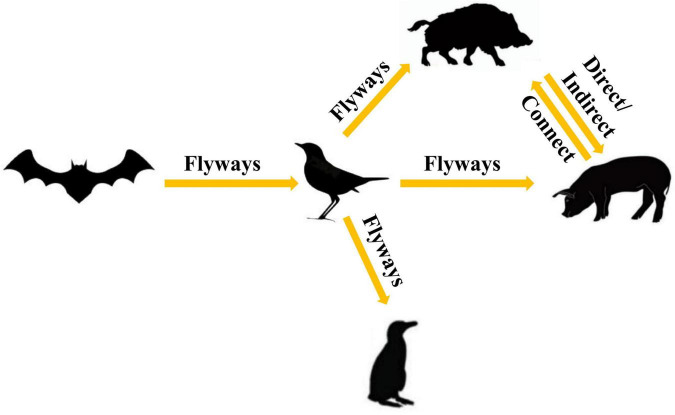
Proposed ecosystem model for the production and cross-species transmission of the PCV3, bat circovirus, bird circovirus, and penguin circovirus.

To further analyze the relationship among the circoviruses in different species, we downloaded other mammalian circoviruses (human circovirus, canine circovirus, fox circovirus, and bat circovirus) and avian circoviruses (duck circovirus, goose circovirus, bird circovirus, and penguin circovirus) to reconstruct the MCC and ML trees using complete genomic sequences (Figure 3 and Supplementary Figure 2). We found that pig circovirus (refers specifically to PCV3) showed high confidence (bootstrap = 95.4%, Supplementary Figure 2) with bird and penguin circoviruses as they were in the same clade, indicating that birds may participate in the spread of circoviruses. Meanwhile, we compared the homology of different circoviruses based on complete genomes and found that the homologies between bat and bird, and bat and penguin circoviruses are 38.3–57.6% and 47.6–56.1%, respectively. The homologies between bat and domestic pig, and bat and wild boar circoviruses are 41–52% and 43.8–49.6%, respectively. On the other hand, the homologies between bird and domestic pig circoviruses, as well as bird and wild boar circoviruses are 50.9–54.1% and 43.2–43.8%, respectively. The homology between penguin and domestic pig circoviruses is 51.3–54.9%. The homology between domestic pig and wild boar circoviruses is 97.6–99.1% (Figure 4). Based on the combined results of the phylogenetic analysis and previous reports, we proposed a new possible way of circovirus transmission, as shown in Figure 5, in which the bat circovirus, being the source, is transmitted to birds; the birds that carry the virus can then spread the circovirus to domestic pigs and/or wild boars, after which epidemics can occur in pigs and wild boars.

**FIGURE 6:**
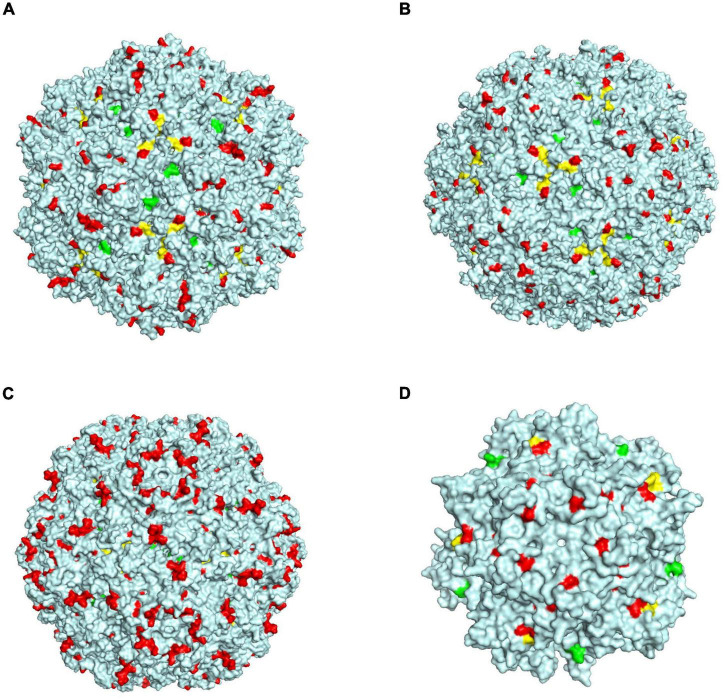
Schematic diagram of the structure simulation of the Cap protein of different circovirus species. **(A)** Green, yellow, and red represent three regions, 78–83, 142–151, and 181–193 of PCV3, respectively. **(B)** Green, yellow, and red represent three regions, 94–99, 162–171, and 198–210 of bat circovirus, respectively. **(C)** Green, yellow, and red represent three regions, 94–99, 161–170, and 209–311 of bird circovirus, respectively. **(D)** Green, yellow, and red represent three regions, 60–65, 125–134, and 174–186 of penguin circovirus, respectively.

When a virus infects a cell, it usually binds to a receptor on the cell surface, after which it enters and infects the cell. The capsid protein of the circovirus is a key protein that binds to cell surface receptors. We compared the homology and differences in the Cap proteins of different species of circoviruses and found that the homology at the amino acid level was not high, but the amino acids in the 78–83, 142–151, and 181–193 amino acid motifs of the PCV3 Cap protein had very high homology (>80%) with those of other circoviruses, including birds, penguins, and bats (Supplementary Figure 3). We performed a structural simulation of the Cap protein and found that these three amino acid motifs are exposed on the outside of the viral capsid surface, as shown in Figure 6. When the surfaces of different species of circoviruses have similar structures, they may bind to similar or the same receptors on the cell surface of different species, thus leading to the transmission of circoviruses within different species. To date, a receptor that can bind to PCV3 has not yet been identified; however, there is a need to consider the possibility of PCV3 using these three amino acid regions with high homology to bind to broad-spectrum receptors on the surface of cells of different species and enter the cells.

### Selection Analysis of Porcine Circovirus Type 3 Cap Protein

Selection pressure analysis can be used to determine whether selection pressure leads to variability. The positive selection sites may explain why PCV3 evades the host immune system and interferes with the long-term prevalence of PCV3 and the evolution of PCV3. We found that three codons (5, 24, and 77) were under positive selection with high confidence, as shown in Table 1. All three positive codons were in the ORF2 coding region and were further confirmed by four different methods (FEL, FUBAR, SLAC, and MEME). Interestingly, according to the predicted epitope (Li et al., 2018), we found that three positive sites were located on the predicted epitope. Furthermore, amino acid 24 of PCV3a (V) was different from that of PCV3b/PCV3c (A), which may lead to the antigenic difference among PCV3a, PCV3b, and PCV3c. However, whether this may be a key point in the variation between PCV3a and PCV3b/3c requires further investigation.

**TABLE 1:** Selection analysis of whole coding sequences of PCV3.

Codon	FEL		SLAC		FUBAR		MEME	
	dN-dS	*p*-value	dN-dS	*p*-value	dN-dS	Post. Pro	W	*p*-value
5	9.366	0.0022	5.37	0.00152	4.313	0.998	9.37	0.00
24	5.866	0.0154	3.69	0.0116	2.394	0.991	7.31	0.01
77	4.229	0.0397	4.90	0.0292	5.454	0.991	6.04	0.02

*The value of p < 0.05 or posterior probability > 0.9 is considered significantly different.*

## Discussion

Porcine circovirus type 3 was first discovered in 2015, and since then, it has been found in many countries, including Asia, America, and Europe (Ha et al., 2020; Saporiti et al., 2020; Assao et al., 2021). However, only a few research data have focused on the phylogeographic distribution and spread of PCV3 (Arruda et al., 2019; Chen et al., 2019; Franzo et al., 2019b; Jiang et al., 2019). Here, we provided new insights into the origin time, evolutionary dynamics, phylogeographic distribution, and mode of spread of PCV3.

The most recent common ancestor is currently the most widely used and accepted method (Gomez-Carballa et al., 2020); however, an insufficient number of sequences or unclear collection time of viral strains may affect the predicted results. In the present study, we collected the sequence information of all PCV3 strains (1996–2021); however, the collection times of most of the strains are not clear. The ambiguity of the collection time of PCV3 strains will have a certain impact on the results of the tMRCA analysis. After examining all combinations of tree models and molecular clocks, we selected the most suitable combination method for reconstructing the PCV3 phylogeny. The tMRCA of PCV3 was estimated to be 1907.53–1923.44 and began to differentiate in the late 1970s (Figures 1A,B), which means that this virus had existed a hundred years before it was identified. In terms of phylogenetic classification, PCV3 has been classified into three clades (Franzo et al., 2020) or three genotypes and several subtypes (Chung et al., 2021). PCV3 is also classified into three clades by a combination of phylogenetic tree and genetic distance in the present study (Figure 1C). According to the time scale, the divergence time of PCV3a was found earlier than that of PCV3b (Figure 1C), thereby indicating that the spread and prevalence of PCV3a were wider than that of PCV3b. In Asia and America, there are both PCV3a and PCV3b strains, with PCV3a being more prevalent. However, PCV3b has not been detected in Europe. These results indicate that the prevalence of PCV3 has a regional specificity. Combined with the time scale and phylogenetic tree node analysis (Figure 1C), it was observed that the divergence time of the two main clades of PCV3 happened during the late 1970s of the world economic recovery. This might be associated with the development of the global economy and trade, thereby promoting the spread of PCV3 around the world during this period. However, the relationship between the PCV3 spread and global trade requires further investigation.

The rate of nucleotide mutation is usually used to measure the speed of viral evolution, and the rapid mutation rate of the virus in the outbreak phase can help the virus better adapt to the new host (Chen et al., 2019). The mutation of the virus is mainly to escape the host’s immune response, and the mutation rate will slow down without the influence of external selective pressure; the most obvious example is the influenza virus (Su et al., 2015). Compared with other types of porcine circoviruses, the substitution rate of PCV3 (4.818 × 10^–4^) is higher than that of PCV1 (1.15 × 10^–5^) but lower than that of PCV2 (1.2 × 10^–3^) (Firth et al., 2009; Cortey and Segales, 2012). One of the reasons for this phenomenon may be the large-scale use of PCV2 vaccines on a global scale since 2006 (Opriessnig et al., 2007; Karuppannan and Opriessnig, 2017), the mutation rate of PCV2 may be significantly faster under the pressure of the antibodies created from the vaccine, thereby accelerating the rate of viral mutation. Although no vaccine is available yet for PCV3, a variety of factors need to be considered in the development and administration of PCV3 vaccines. Importantly, we found that three positive selection sites (5, 24, and 77) of the Cap protein were predicted under positive selection, and site 24 located in the Cap protein was different in PCV3a, PCV3b, and PCV3c. Furthermore, some studies have shown that the selective pressure of the Cap protein of PCV3 is less than that of the cap protein of PCV2 (Franzo et al., 2019b). This change at site 24 is generally considered to be caused by the low virulence of PCV3 or the presence of virus–host co-evolution, which reduces the immune response of infected pigs, but this result is only a speculative result and needs to be verified by immunological research in the future (Chen et al., 2019; Franzo et al., 2019b).

Research on viral traceability is critical for the prevention and control of viral epidemics (Su et al., 2015; Gao, 2018; Franzo et al., 2019b). The survival and spread of the virus require a very suitable environment, such as high temperature and high humidity. From the phylogeographic analysis results, it was found that Malaysia, Thailand, Chile, and Colombia are the four main countries of spread, and Malaysia was the most likely place of PCV3 origin. Interestingly, although the four countries are in Southeast Asia and South America, they are all located within the region 30° south to 30° north latitude. All four countries have tropical or subtropical rainforest climates, with high temperatures and high humidity throughout the year, and this environment is conducive to the spread and mutation of the virus (Hamel et al., 2021; Hattakam et al., 2021; Munoz et al., 2021; Ngim et al., 2021; Sriburin et al., 2021). In subtropical rainforests, PCV3 is not the only prevalent virus; other viruses such as the Zika virus and dengue virus are also prevalent, among others (Hamel et al., 2021; Hattakam et al., 2021; Munoz et al., 2021; Ngim et al., 2021; Sriburin et al., 2021). The spread of the virus may typically be accompanied by economic development and trade. We found that these four countries (Malaysia, Colombia, Thailand, and Chile) have one thing in common: they all have abundant port resources, indicating that these four countries have many trade exchanges with other countries, including livestock exports, which will accelerate the spread of the virus among different countries, resulting in these four countries becoming the main sources of PCV3.

Bats and/or birds are usually the source of multiple viruses or an important intermediate host, including avian influenza, coronavirus, and Zika virus (Dye and Gay, 2003; Bera et al., 2017; Yu et al., 2018; Verdoni et al., 2020; Holmes et al., 2021). We wanted to determine the relationship between PCV3 and Malaysian bat circovirus by combining the phylogeographic and phylogenetic analysis results; however, it was found that so far, no bat circovirus sequence from Malaysia has been deposited in any database. Malaysia has many tropical rainforests, and there are many animals in these rainforests, including bats and birds. In tropical rainforests, the boundaries between wild and domestic animals are unclear, and there are often various contacts among different animals, which increases the risk of transmission among different species (Rendon-Marin et al., 2021). Therefore, researchers need to pay more attention to tropical or subtropical rainforest areas in future epidemic studies.

In this study, the results indicate that PCV3 is more closely related to the bat circovirus clade 1 (Figure 3). Whereas PCV1, PCV2, and PCV4 are shown to be close to the bat circovirus clade 2 (Li et al., 2018; Sun et al., 2021). Bat circovirus clade 1 has been identified in many bat species, including *Plecotus auritus* Linnaeus and *Miniopterus schreibersii* (Li et al., 2021). However, more has been found in bat feces; therefore, it is impossible to determine the specific bat species, but it is certain that bat circovirus clade 1 has been detected in various bat species (Li et al., 2021). Another issue that needs to be addressed in the future is whether the bat circovirus will spill over and cause a huge public health threat. The divergence time between bat circovirus and other circoviruses (porcine circovirus, bird circovirus, and penguin circovirus) is quite different from the divergence time of bat circovirus, which was discovered earlier than other circoviruses. There may still be circovirus lineages that have not been detected. Because bat circoviruses have a long divergence time, virus spillover may occur and transmission may cross within different species during the long-term evolution process (Yu et al., 2018; Holmes et al., 2021). In addition, some mutations that occur in circoviruses may lead to the generation of human-susceptible strains. Circoviruses have already spread in bat colonies for a long time and have been detected in mammals and avians, showing that they have the potential to spread to humans. Bats are considered major reservoirs of new emerging and re-emerging infectious diseases, and the diversity of viruses carried by bats and the dynamic process of recombination between viruses indicate the difficulty to predict the timing of a virus outbreak (Song et al., 2017; Xu et al., 2019). Therefore, it is necessary to establish a global real-time bat virus detection system network to better respond to possible outbreaks.

Numerous studies have demonstrated that the bat circovirus is the origin of the porcine circovirus type 3 (Li et al., 2018; Franzo et al., 2019b; Sun et al., 2021), but these studies have not indicated whether the circovirus is transmitted directly to pigs or whether it is transmitted through an intermediate host. In general, bats are not known to have direct contact with pigs. One or more intermediate hosts may exist in the spread of the circovirus, and PCV3 may spread to pigs after several cross-species transmissions. Phylogenetic analysis suggests that the divergence of avian circoviruses occurs earlier than that of porcine circoviruses (Figure 3). In tropical rainforests, birds are infected with bat circovirus, and then spread the virus to wild boars or directly to domestic pigs through their activities. In this study, we proposed a new transmission route, as shown in Figure 5. In the spread of circoviruses, it was believed that bats have close contact with birds, which causes the transmission of circoviruses from bats to birds. A previous report also showed that PCV3 has close relationship with bird circovirus based on genome composition and codon bias (Franzo et al., 2018). However, how does circovirus spread from birds to pigs? Combined with previous studies, we found that circoviruses such as porcine circovirus and canine circovirus can be detected in feces, which shows that circoviruses can be transmitted through the fecal-oral route (Song et al., 2017; Xu et al., 2019). In other words, birds can simultaneously transmit circoviruses to domestic pigs and wild boars through feces. Therefore, these results showed that circovirus can maintain a certain degree of virulence in feces and can infect other animals through the fecal-oral route. Phylogenetic analysis showed that PCV3 has a close relationship with birds and penguins (Figure 3), which suggests that the transmission mode of circovirus might be similar to that of influenza, in which birds play a crucial role as the intermediate host (Zhou et al., 2018; Karakus et al., 2019; Wang et al., 2020). However, in this cycle, whether the source of porcine circovirus is directly transmitted by birds and/or wild boars is worthy of further analysis. In the structural analysis, the results showed three regions of high homology in the circovirus, and these three regions are located on the surface of the Cap protein (Figure 6), indicating that these regions can be used as potential ligands and can interact with host cells. Moreover, the 181–193 domain is worthy of our attention. It is exposed on the outer surface of the above four circoviruses and has a remarkably high expression level on the outer shell of bird circoviruses (Figure 6C). The receptors bind to it and cross-species transmission occurs. According to previous research, MHC class II proteins can mediate cross-species transmission of the bat origin virus (Li et al., 2021), and in our previous study, after PCV3 infection, we found that the expression of MHC class II protein was associated with SLA-DRB1 and SLADQB expression (Jiang et al., 2020). In addition, PCV3 has been found in many mammals and avian (Zhang et al., 2018; Franzo et al., 2019a; Sun et al., 2019), so it is necessary to study the cross-species situation of PCV3 in future research to rule out whether PCV3 will pose a threat to human health after experiencing multiple cross-species transmissions such as in bats and birds. We also observed that duck, goose, canine, fox, and bat circoviruses are closely related (Figure 3). In summary, circoviruses are widely spread between mammals and poultry and have a relatively close relationship. Therefore, in the future, attention must be paid to the possibility of PCV3 transmission within different species. Transmission of PCV3 in different hosts may be a risk for public safety, similar to the recently identified bat-originating coronavirus found in pigs (Wang et al., 2018; Zhou et al., 2018).

## Conclusion

In conclusion, we also confirmed the bat origin of PCV3, but we further put forward a new point of view that birds are an important intermediate host for the transmission of bat circovirus to pigs, accelerating the global spread of PCV3 in pigs. This mode of spread increases the risk of cross-species transmission, potentially influencing public health. We also proposed that Malaysia is the most likely place of origin and that Thailand, Chile, and Colombia are important transit points for the spread of the virus. Overall, our research provides novel insights into the evolutionary origin, mode of spread, and geographic distribution of PCV3, which should facilitate the planning of effective control strategies for PCV3 infection in the future.

## Data Availability Statement

The raw data supporting the conclusions of this article will be made available by the authors, without undue reservation.

## Author Contributions

YC and JL conceived and designed the experiments. YP, XF, JZ, DW, and JG collected sequence information. CL, YS, TS, and XY sorted the sequences. YC, LH, NZ, YW, and XT analyzed the data. YC, LH, and JL wrote the manuscript. All authors read and approved the final manuscript.

## Conflict of Interest

The authors declare that the research was conducted in the absence of any commercial or financial relationships that could be construed as a potential conflict of interest.

## Publisher’s Note

All claims expressed in this article are solely those of the authors and do not necessarily represent those of their affiliated organizations, or those of the publisher, the editors and the reviewers. Any product that may be evaluated in this article, or claim that may be made by its manufacturer, is not guaranteed or endorsed by the publisher.

## Footnotes

1
https://www.ncbi.nlm.nih.gov/

2
www.swissmodel.org

3
http://www.datamonkey.org/
