# Supplementary figures and images for: Reconstruction of the Evolutionary Origin, Phylodynamics, and Phylogeography of the Porcine Circovirus Type 3
Source: Front Microbiol. 2022 May 18;13:898212. doi: 10.3389/fmicb.2022.898212 (PMC9158500; doi:10.3389/fmicb.2022.898212)

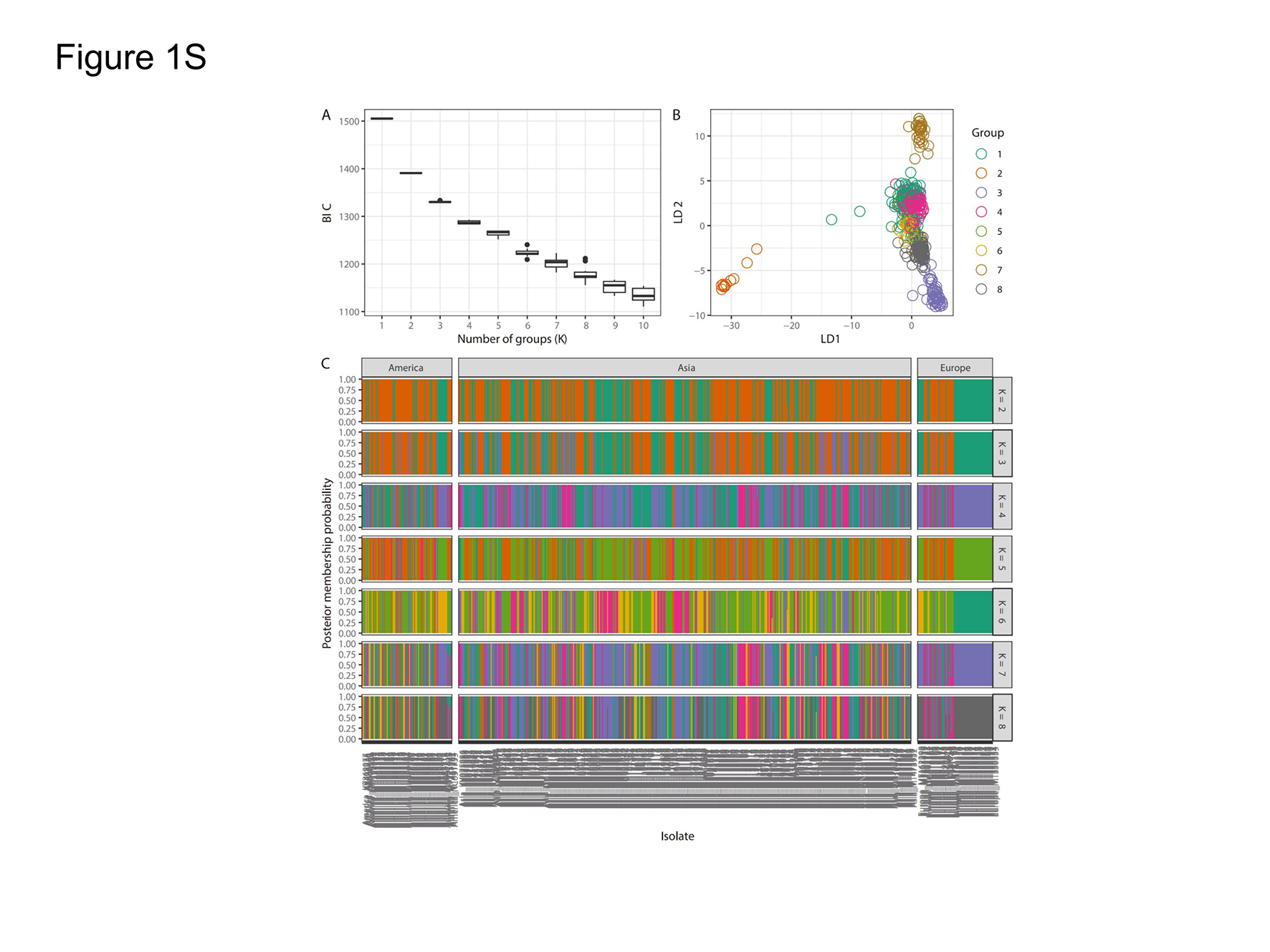

Supplement: Supplementary file 1 [file Image_1.TIF]

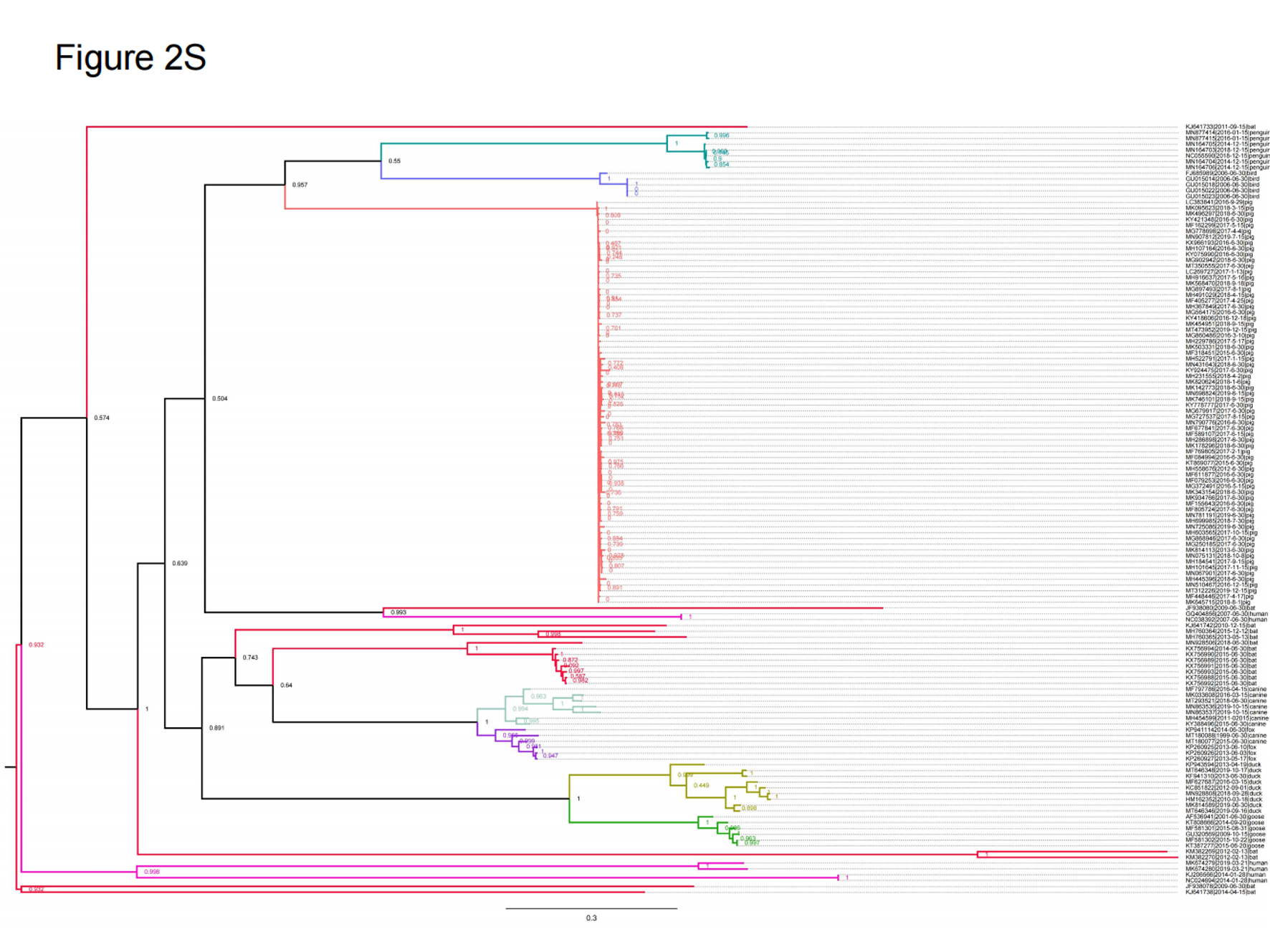

Supplement: Supplementary file 2 [file Image_2.TIF]

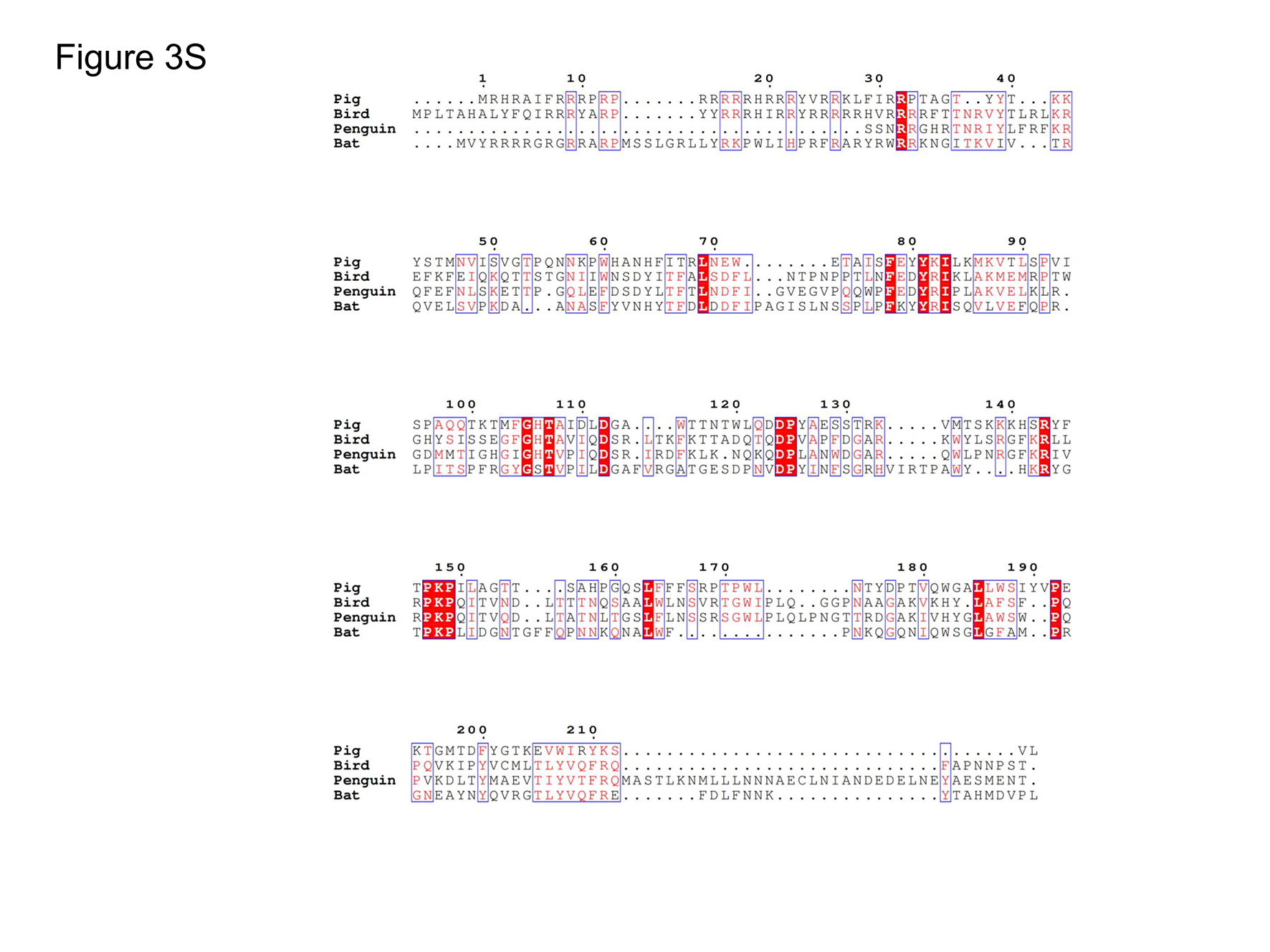

Supplement: Supplementary file 3 [file Image_3.TIF]
