# Supplementary material for: Reconstruction of the Evolutionary Origin, Phylodynamics, and Phylogeography of the Porcine Circovirus Type 3
Source: Front Microbiol. 2022 May 18;13:898212. doi: 10.3389/fmicb.2022.898212 (PMC9158500; doi:10.3389/fmicb.2022.898212)
Supplement: Supplementary file 4 [file Table_1.DOCX]

| **Supplementary Table S1** Referenced PCV3 strains used in phylogenetic analysis | | | |
| --- | --- | --- | --- |
| No. | GenBank Accession No. | Data (Year/Month/Day) | Region |
| 1 | MN725086 | 2019/6/30 | Malaysia |
| 2 | MN725085 | 2019/6/30 | Malaysia |
| 3 | MN725084 | 2019/6/30 | Malaysia |
| 4 | MN725083 | 2019/6/30 | Malaysia |
| 5 | MN725082 | 2019/6/30 | Malaysia |
| 6 | MN725080 | 2018/6/30 | Malaysia |
| 7 | MK820624 | 2018/1/6 | Germany |
| 8 | MK058529 | 2018/9/21 | America |
| 9 | MK058528 | 2018/9/7 | America |
| 10 | MG564175 | 2016/6/30 | China |
| 11 | MG564174 | 2016/6/30 | China |
| 12 | MH603565 | 2017/10/15 | Korea |
| 13 | MH603564 | 2017/6/30 | America |
| 14 | MH603563 | 2017/6/30 | America |
| 15 | MH603562 | 2017/6/30 | America |
| 16 | MH603561 | 2017/6/30 | America |
| 17 | MH603560 | 2017/6/30 | America |
| 18 | MH603559 | 2017/6/30 | America |
| 19 | MH603558 | 2017/6/30 | America |
| 20 | MH603557 | 2017/6/30 | America |
| 21 | MH603556 | 2017/6/30 | America |
| 22 | MH603555 | 2017/6/30 | America |
| 23 | MH603554 | 2017/6/30 | America |
| 24 | MH603553 | 2017/6/30 | America |
| 25 | MH603552 | 2017/6/30 | America |
| 26 | MH603551 | 2017/6/30 | America |
| 27 | MH603550 | 2017/6/30 | America |
| 28 | MH603549 | 2017/6/30 | America |
| 29 | MH603548 | 2017/6/30 | America |
| 30 | MH603547 | 2017/6/30 | America |
| 31 | MH603546 | 2017/6/30 | America |
| 32 | MH603545 | 2017/6/30 | America |
| 33 | MH603544 | 2017/6/30 | America |
| 34 | MH603543 | 2017/6/30 | America |
| 35 | MH603542 | 2017/6/30 | America |
| 36 | MH603541 | 2017/6/30 | America |
| 37 | MH603540 | 2017/6/30 | America |
| 38 | MH603539 | 2017/6/30 | America |
| 39 | MH603538 | 2017/6/30 | America |
| 40 | MH603537 | 2017/6/30 | America |
| 41 | MH603536 | 2017/6/30 | America |
| 42 | MH603535 | 2017/6/30 | America |
| 43 | MH603534 | 2017/6/30 | America |
| 44 | MH603533 | 2017/6/30 | America |
| 45 | MK580468 | 2017/4/15 | China |
| 46 | MK580467 | 2017/6/15 | China |
| 47 | MK580466 | 2018/2/15 | China |
| 48 | MK580465 | 2018/4/15 | China |
| 49 | MH823221 | 2018/8/15 | China |
| 50 | MH823220 | 2018/8/15 | China |
| 51 | MH823219 | 2018/8/15 | China |
| 52 | MG868946 | 2017/6/30 | China |
| 53 | MG868945 | 2017/6/30 | China |
| 54 | MG868944 | 2017/6/30 | China |
| 55 | MG868943 | 2017/6/30 | China |
| 56 | MG868942 | 2017/6/30 | China |
| 57 | MG868941 | 2017/6/30 | China |
| 58 | MG868940 | 2017/6/30 | China |
| 59 | MH327785 | 2018/6/30 | Colombia |
| 60 | MH327784 | 2018/6/30 | Colombia |
| 61 | MG595741 | 2017/8/17 | Hungary |
| 62 | MF079254 | 2016/6/30 | Brazil |
| 63 | MF079253 | 2016/6/30 | Brazil |
| 64 | MG727540 | 2017/8/15 | China |
| 65 | MG727539 | 2017/8/15 | China |
| 66 | MG727538 | 2017/8/15 | China |
| 67 | MG727537 | 2017/8/15 | China |
| 68 | MG550107 | 2017/9/16 | China |
| 69 | MG014376 | 2015/6/30 | Germany |
| 70 | MG014375 | 2015/6/30 | Germany |
| 71 | MG014374 | 2015/6/30 | Germany |
| 72 | MG014373 | 2015/6/30 | Germany |
| 73 | MG014372 | 2015/6/30 | Germany |
| 74 | MG014371 | 2015/6/30 | Germany |
| 75 | MG014370 | 2015/6/30 | Germany |
| 76 | MG014369 | 2015/6/30 | Germany |
| 77 | MG014368 | 2015/6/30 | Germany |
| 78 | MG014367 | 2015/6/30 | Germany |
| 79 | MG014366 | 2015/6/30 | Germany |
| 80 | MG014365 | 2015/6/30 | Germany |
| 81 | MG014364 | 2015/6/30 | Germany |
| 82 | MG014363 | 2015/6/30 | Germany |
| 83 | MG014362 | 2015/6/30 | Germany |
| 84 | KY418606 | 2016/12/18 | China |
| 85 | MT312226 | 2019/12/15 | Taiwan |
| 86 | MT461294 | 2018/6/30 | Colombia |
| 87 | MT461293 | 2015/6/30 | Colombia |
| 88 | MT461292 | 2018/6/30 | Colombia |
| 89 | MT375541 | 2015/6/30 | Colombia |
| 90 | MT375540 | 2018/6/30 | Colombia |
| 91 | MT347692 | 2019/6/30 | Colombia |
| 92 | MN698824 | 2019/6/15 | Korea |
| 93 | MN698823 | 2019/6/15 | Korea |
| 94 | MN698822 | 2019/6/15 | Korea |
| 95 | MN698821 | 2018/6/15 | Korea |
| 96 | MN698820 | 2018/6/15 | Korea |
| 97 | MN698819 | 2018/6/15 | Korea |
| 98 | MN698818 | 2017/6/15 | Korea |
| 99 | MN698817 | 2017/6/15 | Korea |
| 100 | MN698816 | 2017/6/15 | Korea |
| 101 | MN698815 | 2016/6/15 | Korea |
| 102 | MN698814 | 2016/6/15 | Korea |
| 103 | MN698813 | 2016/6/15 | Korea |
| 104 | MN698812 | 2015/6/15 | Korea |
| 105 | MN698811 | 2015/6/15 | Korea |
| 106 | MN698810 | 2015/6/15 | Korea |
| 107 | MN698809 | 2014/6/15 | Korea |
| 108 | MN698808 | 2014/6/15 | Korea |
| 109 | MN698807 | 2014/6/15 | Korea |
| 110 | MN698806 | 2013/6/15 | Korea |
| 111 | MN698805 | 2013/6/15 | Korea |
| 112 | MN698804 | 2013/6/15 | Korea |
| 113 | MN698803 | 2012/6/15 | Korea |
| 114 | MN698802 | 2012/6/15 | Korea |
| 115 | MN698801 | 2012/6/15 | Korea |
| 116 | MN698800 | 2011/6/15 | Korea |
| 117 | MN698799 | 2011/6/15 | Korea |
| 118 | MN698798 | 2011/6/15 | Korea |
| 119 | MN698797 | 2010/6/15 | Korea |
| 120 | MN698796 | 2010/6/15 | Korea |
| 121 | MN698795 | 2010/6/15 | Korea |
| 122 | MN698794 | 2009/6/15 | Korea |
| 123 | MN698793. | 2009/6/15 | Korea |
| 124 | MN698792 | 2009/6/15 | Korea |
| 125 | MT473952 | 2019/12/15 | China |
| 126 | MT473951 | 2019/9/15 | China |
| 127 | MT473950 | 2019/8/15 | China |
| 128 | MT473949 | 2019/9/15 | China |
| 129 | MT473948 | 2018/4/15 | China |
| 130 | MT473947 | 2018/11/15 | China |
| 131 | MT473946 | 2018/7/15 | China |
| 132 | MT473945 | 2018/6/15 | China |
| 133 | MT473944 | 2018/4/15 | China |
| 134 | MT473943 | 2018/4/15 | China |
| 135 | MT473942 | 2018/4/15 | China |
| 136 | MT473941 | 2017/4/15 | China |
| 137 | MT473940 | 2017/11/15 | China |
| 138 | MT473939 | 2017/9/15 | China |
| 139 | MT473938 | 2017/5/15 | China |
| 140 | MT473937 | 2017/5/15 | China |
| 141 | MN790776 | 2016/6/30 | China |
| 142 | MN790775 | 2016/6/30 | China |
| 143 | MN790774 | 2016/6/30 | China |
| 144 | MN507536 | 2018/7/15 | Taiwan |
| 145 | MN510467 | 2016/12/15 | Taiwan |
| 146 | MN510466 | 2017/5/15 | Taiwan |
| 147 | MT104510 | 2018/6/15 | China |
| 148 | MK585353 | 2018/6/30 | Malaysia |
| 149 | MK585352 | 2017/6/30 | Malaysia |
| 150 | MK585351 | 2017/6/30 | Malaysia |
| 151 | MK585350 | 2017/6/30 | Malaysia |
| 152 | MK585347 | 2017/6/30 | Malaysia |
| 153 | MK496297 | 2018/6/30 | America |
| 154 | MK496296 | 2018/6/30 | America |
| 155 | MK496295 | 2018/6/30 | America |
| 156 | MK496294 | 2018/6/30 | America |
| 157 | MK496293 | 2018/6/30 | America |
| 158 | MK496292 | 2018/6/30 | America |
| 159 | MK496291 | 2018/6/30 | America |
| 160 | MK496290 | 2018/6/30 | America |
| 161 | MK496289 | 2018/6/30 | America |
| 162 | MK496288 | 2018/6/30 | America |
| 163 | MK496287 | 2018/6/30 | America |
| 164 | MK496286 | 2018/6/30 | America |
| 165 | MK496285 | 2018/6/30 | America |
| 166 | MK496284 | 2018/6/30 | America |
| 167 | MK496283 | 2018/6/30 | America |
| 168 | MK496282 | 2018/6/30 | America |
| 169 | MK496281 | 2018/6/30 | America |
| 170 | MK496280 | 2018/6/30 | America |
| 171 | MK496279 | 2018/6/30 | America |
| 172 | MK496278 | 2018/6/30 | America |
| 173 | MK496277 | 2018/6/30 | America |
| 174 | MK496276 | 2018/6/30 | America |
| 175 | MK496275 | 2018/6/30 | America |
| 176 | MK496274 | 2018/6/30 | America |
| 177 | MK496273 | 2018/6/30 | America |
| 178 | MK496272 | 2018/6/30 | America |
| 179 | MK496271 | 2018/6/30 | America |
| 180 | MK496270 | 2018/6/30 | America |
| 181 | MK496269 | 2018/6/30 | America |
| 182 | MK105924 | 2018/6/30 | China |
| 183 | MH699985 | 2018/7/30 | Brazil |
| 184 | MH683051 | 2018/6/30 | China |
| 185 | MH177453 | 2017/5/15 | China |
| 186 | MG870097 | 2018/1/27 | China |
| 187 | MG870096 | 2018/1/27 | China |
| 188 | MG870095 | 2018/1/27 | China |
| 189 | MG778698 | 2017/4/4 | China |
| 190 | LC269727 | 2017/1/13 | Japan |
| 191 | MH229786 | 2017/5/17 | Thailand |
| 192 | MH192341 | 2017/9/16 | Mexico |
| 193 | MG546667 | 2016/12/15 | China |
| 194 | MG372491 | 2016/5/15 | China |
| 195 | MG372489 | 2013/7/15 | China |
| 196 | MG372488 | 2006/10/15 | China |
| 197 | MG372487 | 2013/7/15 | China |
| 198 | MG372486 | 2016/8/15 | China |
| 199 | MG372485 | 2016/9/15 | China |
| 200 | MG372484 | 2016/5/15 | China |
| 201 | MG372483 | 2017/5/15 | China |
| 202 | MG253684 | 2017/9/20 | China |
| 203 | MG253683 | 2017/9/20 | China |
| 204 | MG253682 | 2017/9/20 | China |
| 205 | MG253681 | 2017/8/16 | China |
| 206 | MG253680 | 2017/8/16 | China |
| 207 | MG253679 | 2017/9/16 | China |
| 208 | MG253678 | 2017/9/12 | China |
| 209 | MF677841 | 2017/6/30 | China |
| 210 | MF677840 | 2017/6/30 | China |
| 211 | MF677839 | 2017/6/30 | China |
| 212 | MF677838 | 2017/6/30 | China |
| 213 | MF677837 | 2016/6/30 | China |
| 214 | MF677836 | 2016/6/30 | China |
| 215 | MF677835 | 2016/6/30 | China |
| 216 | MG679917 | 2017/6/30 | Russia |
| 217 | MG679916 | 2017/6/30 | Russia |
| 218 | MF448446 | 2017/4/17 | Korea |
| 219 | MF448445 | 2017/4/7 | Korea |
| 220 | MF318453 | 2016/6/30 | China |
| 221 | MF318452 | 2015/6/30 | China |
| 222 | MF318451 | 2015/6/30 | China |
| 223 | MF318450 | 2015/6/30 | China |
| 224 | MF318449 | 2015/6/30 | China |
| 225 | MF318448 | 2016/6/30 | China |
| 226 | MF805724 | 2017/6/30 | Denmark |
| 227 | MF805723 | 2017/6/30 | Denmark |
| 228 | MF805722 | 2016/6/30 | Italy |
| 229 | MF805721 | 2016/6/30 | Italy |
| 230 | MF805720 | 2017/6/30 | Spain |
| 231 | MF805719 | 2017/6/30 | Italy |
| 232 | MG310152 | 2017/7/15 | Thailand |
| 233 | KY421348 | 2016/6/30 | China |
| 234 | KY421347 | 2016/6/30 | China |
| 235 | MF069116 | 2016/6/30 | China |
| 236 | MF069115 | 2016/12/15 | China |
| 237 | MF405277 | 2017/4/25 | China |
| 238 | MF405274 | 2017/4/20 | China |
| 239 | MF405273 | 2017/4/15 | China |
| 240 | MF405272 | 2017/4/12 | China |
| 241 | MF589107 | 2017/6/15 | China |
| 242 | MF589106 | 2016/2/15 | China |
| 243 | MF589105 | 2016/2/15 | China |
| 244 | MF589104 | 2015/8/15 | China |
| 245 | MF589103 | 2015/4/15 | China |
| 246 | MF589102 | 2016/6/15 | China |
| 247 | MF162299 | 2017/5/15 | Italy |
| 248 | MF162298 | 2017/5/15 | Italy |
| 249 | KY075994 | 2016/6/30 | China |
| 250 | KY075993 | 2016/6/30 | China |
| 251 | KY075992 | 2016/6/30 | China |
| 252 | KY075991 | 2016/6/30 | China |
| 253 | KY075990 | 2016/6/30 | China |
| 254 | KY075989 | 2016/6/30 | China |
| 255 | KY075988 | 2016/6/30 | China |
| 256 | KY075987 | 2016/6/30 | China |
| 257 | KY075986 | 2016/6/30 | China |
| 258 | KY354039 | 2016/6/30 | China |
| 259 | KY354038 | 2016/6/30 | China |
| 260 | MN781192 | 2019/6/30 | Italy |
| 261 | MN781191 | 2019/6/30 | Italy |
| 262 | MN781190 | 2019/6/30 | Italy |
| 263 | MN781189 | 2019/6/30 | Italy |
| 264 | MN781188 | 2019/6/30 | Italy |
| 265 | MN781187 | 2018/6/30 | Italy |
| 266 | MN788153 | 2017/6/30 | China |
| 267 | MN788152 | 2017/6/30 | China |
| 268 | MN788151 | 2016/6/30 | China |
| 269 | MN788150 | 2017/6/30 | China |
| 270 | MN788149 | 2018/6/30 | China |
| 271 | MN788148 | 2018/6/30 | China |
| 272 | MN788147 | 2017/6/30 | China |
| 273 | MN788146 | 2016/6/30 | China |
| 274 | MN788145 | 2017/6/30 | China |
| 275 | MN788144 | 2017/6/30 | China |
| 276 | MN788143 | 2017/6/30 | China |
| 277 | MN788142 | 2016/6/30 | China |
| 278 | MN788141 | 2016/6/30 | China |
| 279 | MN788140 | 2017/6/30 | China |
| 280 | MN788139 | 2017/6/30 | China |
| 281 | MN788138 | 2017/6/30 | China |
| 282 | MN788137 | 2019/6/30 | China |
| 283 | MN788136 | 2017/6/30 | China |
| 284 | MN788135 | 2017/6/30 | China |
| 285 | MN788134 | 2019/6/30 | China |
| 286 | MN788133 | 2016/6/30 | China |
| 287 | MN788132 | 2019/6/30 | China |
| 288 | MN788131 | 2016/6/30 | China |
| 289 | MN788130 | 2016/6/30 | China |
| 290 | MN788129 | 2016/6/30 | China |
| 291 | MN788128 | 2019/6/30 | China |
| 292 | MN788127 | 2016/6/30 | China |
| 293 | MN788126 | 2017/6/30 | China |
| 294 | MN788125 | 2017/6/30 | China |
| 295 | MN788124 | 2018/6/30 | China |
| 296 | MN788123 | 2018/6/30 | China |
| 297 | MT350555 | 2017/6/30 | Spain |
| 298 | MT350554 | 2017/6/30 | Spain |
| 299 | MT350553 | 2017/6/30 | Spain |
| 300 | MT350552 | 2017/6/30 | Spain |
| 301 | MT350551 | 2017/6/30 | Spain |
| 302 | MT350550 | 2017/6/30 | Spain |
| 303 | MT350549 | 2017/6/30 | Spain |
| 304 | MT350548 | 2017/6/30 | Spain |
| 305 | MT350547 | 2017/6/30 | Spain |
| 306 | MT350546 | 2017/6/30 | Spain |
| 307 | MT350545 | 2017/6/30 | Spain |
| 308 | MT350544 | 2017/6/30 | Spain |
| 309 | MT350543 | 2017/6/30 | Spain |
| 310 | MT350542 | 2017/6/30 | Spain |
| 311 | MT350541 | 2017/6/30 | Spain |
| 312 | MT350540 | 2017/6/30 | Spain |
| 313 | MT350539 | 2017/6/30 | Spain |
| 314 | MT350538 | 2017/6/30 | Spain |
| 315 | MT350537 | 2017/6/30 | Spain |
| 316 | MT350536 | 2017/6/30 | Spain |
| 317 | MT350535 | 2017/6/30 | Spain |
| 318 | MT350534 | 2017/6/30 | Spain |
| 319 | MT350533 | 2017/6/30 | Spain |
| 320 | MT350532 | 2017/6/30 | Spain |
| 321 | MT350531 | 2017/6/30 | Spain |
| 322 | MT350530 | 2017/6/30 | Spain |
| 323 | MT350529 | 2017/6/30 | Spain |
| 324 | MT350528 | 2017/6/30 | Spain |
| 325 | MT350527 | 2017/6/30 | Spain |
| 326 | MT350526 | 2017/6/30 | Spain |
| 327 | MT350525 | 2017/6/30 | Spain |
| 328 | MT350524 | 2017/6/30 | Spain |
| 329 | MT350523 | 2017/6/30 | Spain |
| 330 | MT350522 | 2017/6/30 | Spain |
| 331 | MT350521 | 2017/6/30 | Spain |
| 332 | MT350520 | 2017/6/30 | Spain |
| 333 | MT350519 | 2017/6/30 | Spain |
| 334 | MT350518 | 2017/6/30 | Spain |
| 335 | MT350517 | 2017/6/30 | Spain |
| 336 | MN067901 | 2017/6/30 | China |
| 337 | MN067900 | 2017/6/30 | China |
| 338 | MN067899 | 2018/6/30 | China |
| 339 | MN067898 | 2018/6/30 | China |
| 340 | MN067897 | 2017/6/30 | China |
| 341 | MN067896 | 2017/6/30 | China |
| 342 | MN031988 | 2016/12/15 | China |
| 343 | MN431644 | 2018/6/30 | China |
| 344 | MN431643 | 2018/6/30 | China |
| 345 | MN431642 | 2018/6/30 | China |
| 346 | MN431641 | 2018/6/30 | China |
| 347 | MN431640 | 2018/6/30 | China |
| 348 | MN431639 | 2018/6/30 | China |
| 349 | MN431638 | 2018/6/30 | China |
| 350 | MN431637 | 2018/6/30 | China |
| 351 | MN907812 | 2019/7/15 | Chile |
| 352 | MN075134 | 2018/12/14 | China |
| 353 | MN075133 | 2018/9/5 | China |
| 354 | MN075132 | 2018/9/21 | China |
| 355 | MN075131 | 2018/10/8 | China |
| 356 | MN075130 | 2018/5/16 | China |
| 357 | MN075129 | 2018/4/13 | China |
| 358 | MN075128 | 2018/1/2 | China |
| 359 | MH184542 | 2017/9/15 | China |
| 360 | MH184541 | 2017/9/15 | China |
| 361 | MH184540 | 2017/9/15 | China |
| 362 | MH184539 | 2017/9/15 | China |
| 363 | MH184538 | 2017/9/15 | China |
| 364 | MH184537 | 2017/9/15 | China |
| 365 | MH184536 | 2017/9/15 | China |
| 366 | MH184535 | 2017/9/15 | China |
| 367 | MH184534 | 2017/9/15 | China |
| 368 | MH184533 | 2017/9/15 | China |
| 369 | MK452768 | 2017/12/12 | Korea |
| 370 | MK934768 | 2017/6/30 | India |
| 371 | MK934767 | 2017/6/30 | India |
| 372 | MK934766 | 2017/6/30 | India |
| 373 | MK934765 | 2017/6/30 | India |
| 374 | MF072716 | 2017/2/3 | China |
| 375 | MK746104 | 2018/9/15 | China |
| 376 | MK746103 | 2019/1/15 | China |
| 377 | MK746102 | 2018/9/15 | China |
| 378 | MK746101 | 2018/9/15 | China |
| 379 | MK746100 | 2018/9/15 | China |
| 380 | MK746099 | 2018/8/15 | China |
| 381 | MK746098 | 2018/8/15 | China |
| 382 | MK814116 | 2006/6/30 | China |
| 383 | MK814114 | 2013/6/30 | China |
| 384 | MK814113 | 2013/6/30 | China |
| 385 | MK656956 | 2018/5/15 | China |
| 386 | MK645719 | 2018/8/1 | Brazil |
| 387 | MK645718 | 2018/8/1 | Brazil |
| 388 | MK645717 | 2018/8/1 | Brazil |
| 389 | MK645716 | 2018/8/1 | Brazil |
| 390 | MK645715 | 2018/8/1 | Brazil |
| 391 | MK568470 | 2018/9/18 | America |
| 392 | MK568469 | 2018/9/18 | America |
| 393 | MK178322 | 2018/6/30 | China |
| 394 | MK178321 | 2018/6/30 | China |
| 395 | MK178320 | 2018/6/30 | China |
| 396 | MK178319 | 2018/6/30 | China |
| 397 | MK178318 | 2018/6/30 | China |
| 398 | MK178317 | 2018/6/30 | China |
| 399 | MK178316 | 2018/6/30 | China |
| 400 | MK178315 | 2018/6/30 | China |
| 401 | MK178314 | 2018/6/30 | China |
| 402 | MK178313 | 2018/6/30 | China |
| 403 | MK178312 | 2018/6/30 | China |
| 404 | MK178311 | 2018/6/30 | China |
| 405 | MK178310 | 2018/6/30 | China |
| 406 | MK178309 | 2018/6/30 | China |
| 407 | MK178308 | 2018/6/30 | China |
| 408 | MK178307 | 2018/6/30 | China |
| 409 | MK178306 | 2018/6/30 | China |
| 410 | MK178305 | 2018/6/30 | China |
| 411 | MK178304 | 2018/6/30 | China |
| 412 | MK178303 | 2018/6/30 | China |
| 413 | MK178302 | 2018/6/30 | China |
| 414 | MK178301 | 2018/6/30 | China |
| 415 | MK178300 | 2018/6/30 | China |
| 416 | MK178299 | 2018/6/30 | China |
| 417 | MK178298 | 2018/6/30 | China |
| 418 | MK178297 | 2018/6/30 | China |
| 419 | MG902942 | 2018/6/30 | China |
| 420 | MG902941 | 2018/6/30 | China |
| 421 | MG902940 | 2018/6/30 | China |
| 422 | MG902939 | 2018/6/30 | China |
| 423 | MK343155 | 2018/6/30 | China |
| 424 | MK343154 | 2018/6/30 | China |
| 425 | MK284236 | 2018/6/30 | China |
| 426 | MK178296 | 2018/6/30 | China |
| 427 | MK178295 | 2018/6/30 | China |
| 428 | MK178294 | 2018/6/30 | China |
| 429 | MK178293 | 2018/6/30 | China |
| 430 | MK178292 | 2018/6/30 | China |
| 431 | MK178291 | 2017/6/30 | China |
| 432 | MK178290 | 2017/6/30 | China |
| 433 | MK178289 | 2018/6/30 | China |
| 434 | MK178288 | 2018/6/30 | China |
| 435 | MK178287 | 2018/6/30 | China |
| 436 | MK178286 | 2017/6/30 | China |
| 437 | MK178285 | 2018/6/30 | China |
| 438 | MK178284 | 2018/6/30 | China |
| 439 | MK178283 | 2018/6/30 | China |
| 440 | MK178282 | 2018/6/30 | China |
| 441 | MK178281 | 2018/6/30 | China |
| 442 | MK178280 | 2018/6/30 | China |
| 443 | MK117051 | 2018/6/30 | Serbia |
| 444 | MK503331 | 2018/6/30 | Korea |
| 445 | MK347417 | 2018/6/30 | China |
| 446 | MK347416 | 2017/6/30 | China |
| 447 | MK347415 | 2016/6/30 | China |
| 448 | MK347414 | 2017/6/30 | China |
| 449 | MH231555 | 2018/4/2 | Korea |
| 450 | MH231554 | 2018/1/31 | Korea |
| 451 | MH231553 | 2018/1/31 | Korea |
| 452 | MH231552 | 2018/1/17 | Korea |
| 453 | MK000387 | 2018/1/5 | China |
| 454 | MK095625 | 2018/8/15 | China |
| 455 | MK095624 | 2018/4/15 | China |
| 456 | MK095623 | 2018/3/15 | China |
| 457 | MK095622 | 2018/3/15 | China |
| 458 | MK095621 | 2018/1/15 | China |
| 459 | MK095620 | 2018/1/15 | China |
| 460 | MK340753 | 2018/7/15 | China |
| 461 | MH916639 | 2017/5/16 | China |
| 462 | MH916638 | 2017/5/16 | China |
| 463 | MH916637 | 2017/5/16 | China |
| 464 | MH916636 | 2017/5/16 | China |
| 465 | MH916635 | 2017/5/16 | China |
| 466 | MK454953 | 2017/9/15 | China |
| 467 | MK454952 | 2018/9/15 | China |
| 468 | MK454951 | 2018/9/15 | China |
| 469 | MH547276 | 2018/3/15 | China |
| 470 | MH107164 | 2016/6/30 | China |
| 471 | MH107163 | 2016/6/30 | China |
| 472 | MH107162 | 2016/6/30 | China |
| 473 | MH107161 | 2016/6/30 | China |
| 474 | MH445396 | 2018/6/30 | China |
| 475 | MH445395 | 2018/6/30 | China |
| 476 | MH445394 | 2018/6/30 | China |
| 477 | MH445393 | 2018/6/30 | China |
| 478 | MH367850 | 2017/6/30 | China |
| 479 | MH367849 | 2017/6/30 | China |
| 480 | MH367848 | 2017/6/30 | China |
| 481 | MH367847 | 2017/6/30 | China |
| 482 | MH367846 | 2017/6/30 | China |
| 483 | MH367845 | 2017/6/30 | China |
| 484 | MK142773 | 2018/6/30 | China |
| 485 | MK142772 | 2017/6/30 | China |
| 486 | MK142771 | 2016/6/30 | China |
| 487 | MH491030 | 2018/2/15 | China |
| 488 | MH491029 | 2018/4/15 | China |
| 489 | MH491028 | 2017/10/15 | China |
| 490 | MH491027 | 2017/8/15 | China |
| 491 | MH491026 | 2017/6/15 | China |
| 492 | MH491025 | 2017/2/15 | China |
| 493 | MH491024 | 2018/4/15 | China |
| 494 | MH491023 | 2018/3/15 | China |
| 495 | MH491022 | 2018/3/15 | China |
| 496 | MH491021 | 2018/2/15 | China |
| 497 | MH491020 | 2017/12/15 | China |
| 498 | MH491019 | 2017/5/15 | China |
| 499 | MH491018 | 2017/2/15 | China |
| 500 | MH491017 | 2018/3/15 | China |
| 501 | MH491016 | 2018/1/15 | China |
| 502 | MH286898 | 2017/6/30 | China |
| 503 | MH410564 | 2016/1/14 | China |
| 504 | MH410563 | 2016/1/13 | China |
| 505 | MH410562 | 2015/6/10 | China |
| 506 | MH410561 | 2016/1/3 | China |
| 507 | MG897494 | 2017/8/1 | China |
| 508 | MG897493 | 2017/8/1 | China |
| 509 | MG897492 | 2017/8/1 | China |
| 510 | MG897491 | 2017/8/1 | China |
| 511 | MG897490 | 2017/8/1 | China |
| 512 | MG897489 | 2017/8/1 | China |
| 513 | MG897488 | 2017/8/1 | China |
| 514 | MG897487 | 2017/8/1 | China |
| 515 | MG897486 | 2017/8/1 | China |
| 516 | MG897485 | 2017/8/1 | China |
| 517 | MG897484 | 2017/8/1 | China |
| 518 | MG897483 | 2017/8/1 | China |
| 519 | MG897482 | 2017/8/1 | China |
| 520 | MG897481 | 2017/8/1 | China |
| 521 | MG897480 | 2017/8/1 | China |
| 522 | MG897479 | 2017/8/1 | China |
| 523 | MG897478 | 2017/8/1 | China |
| 524 | MG897477 | 2017/8/1 | China |
| 525 | MG897476 | 2017/8/1 | China |
| 526 | MG897475 | 2017/8/1 | China |
| 527 | MG897474 | 2017/8/1 | China |
| 528 | MG897473 | 2017/8/1 | China |
| 529 | MH101645 | 2017/11/15 | China |
| 530 | MH558676 | 2012/6/30 | Italy |
| 531 | MG947596 | 2017/6/30 | China |
| 532 | MH277119 | 2018/6/30 | China |
| 533 | MH277118 | 2018/6/30 | China |
| 534 | MH277117 | 2018/6/30 | China |
| 535 | MH277116 | 2018/6/30 | China |
| 536 | MH277115 | 2018/6/30 | China |
| 537 | MH277114 | 2018/6/30 | China |
| 538 | MH277113 | 2018/6/30 | China |
| 539 | MH277112 | 2018/6/30 | China |
| 540 | MH277111 | 2018/6/30 | China |
| 541 | MH277110 | 2018/6/30 | China |
| 542 | MH277109 | 2018/6/30 | China |
| 543 | MH277108 | 2018/6/30 | China |
| 544 | MH277107 | 2018/6/30 | China |
| 545 | MH522791 | 2017/1/15 | China |
| 546 | LC383841 | 2016/9/29 | Japan |
| 547 | LC383840 | 2016/1/25 | Japan |
| 548 | MG696866 | 2017/3/15 | China |
| 549 | MH192340 | 2017/7/2 | Mexico |
| 550 | MH121060 | 2017/3/30 | China |
| 551 | MH607133 | 2018/2/15 | China |
| 552 | NC031753 | 2015/6/30 | America |
| 553 | MG860486 | 2016/3/10 | China |
| 554 | MF593110 | 2016/6/30 | China |
| 555 | MG650176 | 1996/6/30 | China |
| 556 | MG650175 | 1998/6/30 | China |
| 557 | MG650174 | 1998/6/30 | China |
| 558 | MG650173 | 1998/6/30 | China |
| 559 | MG650172 | 1998/6/30 | China |
| 560 | MG250187 | 2017/6/30 | China |
| 561 | MG250186 | 2017/6/30 | China |
| 562 | MG250185 | 2017/6/30 | China |
| 563 | MG250184 | 2017/6/30 | China |
| 564 | MG250183 | 2017/6/30 | China |
| 565 | MG250182 | 2017/6/30 | China |
| 566 | MG250181 | 2017/6/30 | China |
| 567 | MG250180 | 2017/6/30 | China |
| 568 | MG250179 | 2016/6/30 | China |
| 569 | MG250178 | 2017/6/30 | China |
| 570 | MG250177 | 2017/6/30 | China |
| 571 | MG250176 | 2017/6/30 | China |
| 572 | MF069252 | 2017/6/30 | China |
| 573 | MF063071 | 2016/6/30 | Korea |
| 574 | MF063070 | 2017/6/30 | Korea |
| 575 | KY924475 | 2017/6/30 | China |
| 576 | KY924474 | 2016/6/30 | China |
| 577 | KY924473 | 2017/6/30 | China |
| 578 | KY924472 | 2017/6/30 | China |
| 579 | MF611878 | 2017/6/30 | Korea |
| 580 | MG765473 | 2004/6/30 | Sweden |
| 581 | KY753913 | 2016/6/30 | China |
| 582 | KY753912 | 2016/6/30 | China |
| 583 | KY753911 | 2016/6/30 | China |
| 584 | MF155643 | 2016/6/30 | China |
| 585 | MF155642 | 2016/6/30 | China |
| 586 | MF155641 | 2016/6/30 | China |
| 587 | MF405275 | 2017/4/22 | China |
| 588 | MF405271 | 2017/3/7 | China |
| 589 | MF084994 | 2016/6/30 | China |
| 590 | MF611877 | 2016/6/30 | Korea |
| 591 | MF611876 | 2016/6/30 | Korea |
| 592 | KY865243 | 2016/7/15 | China |
| 593 | KY865242 | 2016/7/15 | China |
| 594 | KY778777 | 2017/6/30 | China |
| 595 | KY778776 | 2017/6/30 | China |
| 596 | KY996345 | 2016/6/30 | Korea |
| 597 | KY996344 | 2016/6/30 | Korea |
| 598 | KY996343 | 2016/6/30 | Korea |
| 599 | KY996342 | 2016/6/30 | Korea |
| 600 | KY996341 | 2016/6/30 | Korea |
| 601 | KY996340 | 2016/6/30 | Korea |
| 602 | KY996339 | 2016/6/30 | Korea |
| 603 | KY996338 | 2016/6/30 | Korea |
| 604 | KY996337 | 2016/6/30 | Korea |
| 605 | KX966193 | 2016/6/30 | America |
| 606 | KX898030 | 2016/6/30 | America |
| 607 | KX778720 | 2015/6/30 | America |
| 608 | KX458235 | 2015/6/30 | America |
| 609 | KT869077 | 2015/6/30 | America |
| 610 | MF769811 | 2017/2/1 | China |
| 611 | MF769810 | 2017/2/1 | China |
| 612 | MF769809 | 2017/2/1 | China |
| 613 | MF769808 | 2017/2/1 | China |
| 614 | MF769807 | 2017/2/1 | China |
| 615 | MF769806 | 2017/2/1 | China |
| 616 | MF769805 | 2017/2/1 | China |
