# Supplementary material for: Reconstruction of the Evolutionary Origin, Phylodynamics, and Phylogeography of the Porcine Circovirus Type 3
Source: Front Microbiol. 2022 May 18;13:898212. doi: 10.3389/fmicb.2022.898212 (PMC9158500; doi:10.3389/fmicb.2022.898212)
Supplement: Supplementary file 5 [file Table_2.DOCX]

| **Supplementary Table S2** Bayes Factor (BF) and Posterior Probability (PP) tests for PCV3 transmission links in worldwide. | | | | | | |
| --- | --- | --- | --- | --- | --- | --- |
| From | To | | Mean Transition Rate | | Bayes Factory (BF>3) | Posterior Probability (PP>0.3) |
| Malaysia | | Chile | | 0.9828 | 91370.49 | 1 |
| Malaysia | | Thailand | | 1.0035 | 91370.49 | 1 |
| Malaysia | | China | | 0.9252 | 91370.49 | 1 |
| Malaysia | | Russia | | 0.9958 | 13037.49 | 0.998599 |
| Malaysia | | Germany | | 0.9243 | 11405.3 | 0.998398 |
| Malaysia | | Colombia | | 0.9588 | 1432.32 | 0.987387 |
| Thailand | | Colombia | | 0.9293 | 648.7753 | 0.972573 |
| Malaysia | | Sweden | | 1.0228 | 296.8377 | 0.941942 |
| Malaysia | | Japan | | 0.9709 | 266.4042 | 0.935736 |
| China | | Denmark | | 0.6158 | 190.3541 | 0.912312 |
| Chile | | Mexico | | 0.9653 | 180.3752 | 0.907908 |
| Malaysia | | America | | 0.9250 | 89.3468 | 0.83003 |
| Colombia | | America | | 0.9391 | 46.65688 | 0.798318 |
| Malaysia | | Korea | | 0.9640 | 29.65189 | 0.698418 |
| Malaysia | | Serbia | | 0.9975 | 21.33482 | 0.638338 |
| Malaysia | | Hungary | | 1.0337 | 20.87616 | 0.622933 |
| Malaysia | | Italy | | 0.9786 | 14.09983 | 0.595235 |
| Thailand | | Hungary | | 0.9490 | 13.26085 | 0.56022 |
| Malaysia | | India | | 1.0253 | 9.936503 | 0.47952 |
| Germany | | Brazil | | 0.9455 | 9.313851 | 0.43337 |
| Malaysia | | Spain | | 0.9554 | 7.476306 | 0.43009 |
| Malaysia | | Brazil | | 0.9916 | 6.700879 | 0.398068 |
| Chile | | Korea | | 1.0064 | 5.938575 | 0.375045 |
| Colombia | | Spain | | 1.0297 | 4.614146 | 0.371401 |
| Chile | | Spain | | 1.0200 | 4.47713 | 0.346597 |
| Colombia | | Japan | | 0.9546 | 3.950486 | 0.327578 |
| Japan | | Brazil | | 0.9932 | 3.535955 | 0.321962 |
| Colombia | | Serbia | | 1.0162 | 3.106473 | 0.305145 |
